# Adult Hippocampal Neurogenesis: Regulation and Possible Functional and Clinical Correlates

**DOI:** 10.3389/fnana.2018.00044

**Published:** 2018-06-05

**Authors:** Pedro Baptista, José P. Andrade

**Affiliations:** ^1^Unit of Anatomy, Department of Biomedicine, Faculty of Medicine of University of Porto, Porto, Portugal; ^2^Center of Health Technology and Services Research (CINTESIS), Faculty of Medicine of University of Porto, Porto, Portugal

**Keywords:** neurogenesis, man, hippocampal formation, dentate gyrus, cognition, memory, depression

## Abstract

The formation of new neurons in the adult central nervous system (CNS) has been recognized as one of the major findings in neuroanatomical research. The hippocampal formation (HF), one of the main targets of these investigations, holds a neurogenic niche widely recognized among several mammalian species and whose existence in the human brain has sparked controversy and extensive debate. Many cellular features from this region emphasize that hippocampal neurogenesis suffers changes with normal aging and, among regulatory factors, physical exercise and chronic stress provoke opposite effects on cell proliferation, maturation and survival. Considering the numerous functions attributable to the HF, increasing or decreasing the integration of new neurons in the delicate neuronal network might be significant for modulation of cognition and emotion. The role that immature and mature adult-born neurons play in this circuitry is still mostly unknown but it could prove fundamental to understand hippocampal-dependent cognitive processes, the pathophysiology of depression, and the therapeutic effects of antidepressant medication in modulating behavior and mental health.

## Introduction

Neurogenesis is the biological process through which new neurons are formed (Altman and Das, 1965). Neural stem cells proliferate, migrate and differentiate during embryogenesis into mature neurons that will eventually form the central nervous system (CNS; Angevine, 1965). Although the original work of Santiago Ramón y Cajal stated that a similar process of neuron proliferation in the mature brain was inexistent, studies on adult neurogenesis remained central in the scientific community. The paradigm finally changed with cumulative evidence from the second half of the 20th century, which saw the birth of new methods and techniques that allowed the discovery of adult-born neurons in the brains of rodents (Altman, 1962), primates (Gould et al., 1998) and humans (Eriksson et al., 1998). This raised relevant questions on the ultimate significance and impact of postdevelopmental neurogenesis, especially when only three distinct and specific neurogenic areas were mapped in the human brain (Ernst and Frisen, 2015): the hippocampal formation (HF), the subventricular zone (SVZ) of the lateral ventricles and the striatum.

Here, the discussion will be centered in the HF, as it remains the most likely neurogenic niche to play a significant role in brain function (Spalding et al., 2013). Although beyond the scope of this review, the SVZ and the striatum could also display remarkable features and there is still much to be explored in these brain areas.

## Adult Hippocampal Neurogenesis in Humans

The original study that established the presence of neurogenesis in the adult human brain used a thymidine analog incorporated into proliferating cells during DNA synthesis, bromodeoxyuridine (BrdU), to demonstrate proliferation, differentiation, and survival of cells in the dentate gyrus (DG), in the HF (Eriksson et al., 1998). In the study, both the granular layer of the DG and the adjacent subgranular zone (SGZ) presented labeled cells, indicating the presence of recently-generated neurons (Eriksson et al., 1998). This phenomenon was apparently restricted to the DG, as no other hippocampal region demonstrated such activity in man (Eriksson et al., 1998; Knoth et al., 2010). This occurs similarly to other mammals (Kempermann, 2012), making the SGZ widely recognized as neurogenic (Altman and Das, 1965) and standing as the putative source of neural progenitors for the granular layer in primates (Kohler et al., 2011). Using marmoset monkeys injected with BrdU (Gould et al., 1998), results after 2 h showed labeled cells located in the SGZ with the morphological characteristics of granule cells precursors. After a 3-week post-BrdU injection period, most labeled cells were now located in the granular layer and exhibited mature phenotype. The same pattern of migration and differentiation is seen in rodents and indicates that neurogenesis *per se* is a property of the SGZ, not the granular layer (Kohler et al., 2011).

### Extent of the Neurogenic Activity

Recent data from human post-operative and post mortem samples of the HF suggest a very different scenario from the initial study. Using immunohistochemistry to identify markers of proliferation and immature/young neurons, like Ki67, doublecortin (DCX) or polysialylated neural cell adhesion molecule (PSA-NCAM), it was found that either the SGZ proliferative activity in the adult is similar to the regular parenchyma, reflecting microglia (Dennis et al., 2016), or that the SGZ is actually never formed as a consolidated germinal structure at any point during fetal or postnatal development of the human brain, unlike rodents and other primates (Sorrells et al., 2018). The direct conclusion for both findings was the virtual absence of neurogenesis in the adult human HF.

However, these results not only disagree on the existence of a germinal SGZ in humans as they also conflict with positive reports of adult neurogenesis using the same endogenous markers (Knoth et al., 2010; Boldrini et al., 2018). For instance, using the whole human HF and a more unbiased stereology-based approach, thousands of neural progenitor cells and double DCX-positive and PSA-NCAM-positive immature neurons were found in the adult SGZ and granular layer (Boldrini et al., 2018). In this study, a “psychological autopsy” was performed to exclude brain specimens from donors potentially affected by neuropsychiatric diseases or chronic illnesses, which could affect neurogenesis and lead to potentially compromised results in immunohistochemistry analysis (Boldrini et al., 2018). Nonetheless, inconsistency between studies could also arise from the methodology itself. Although BrdU staining for new neurons might be less sensitive than DCX and may produce false-positive results (Sorrells et al., 2018), endogenous markers may be difficult to evaluate in adult brain tissue and may suffer rapid molecular changes if not quickly preserved and correctly fixated post mortem (Knoth et al., 2010; Boldrini et al., 2018). The apparent loss of neurons might instead reflect modification in marker expression. Additionally, the assumption that these markers are evolutionary conserved, and therefor identical in rodents and humans in representing the same cell types, is not yet entirely secure (Knoth et al., 2010; Göritz and Frisén, 2012; Snyder, 2018).

Despite the inherent controversy of using indirect ways to study neurogenesis, more direct methods remain scarce. Neural precursors have been identified and directly isolated from the adult human HF and shown to mature as neurons *in vitro* (Roy et al., 2000). However, this selective extraction lacks quantitative analysis, therefore leaving doubts on the extent of cell production (Eriksson et al., 1998; Boldrini et al., 2018). Thus, even when successful, direct approaches normally yield limited results and require complementary studies. For instance, some reports focusing on neuroblast counts have revealed small numbers of cells after the first postnatal year (Knoth et al., 2010), which in fact could anticipate an almost irrelevant extent of neurogenesis in the DG during adulthood (Dennis et al., 2016; Sorrells et al., 2018). On the other hand, carbon-14 assessment, which allowed retrospective birth dating of neurons through quantification of the isotope concentration in genomic DNA, demonstrated that even small numbers of neuronal precursors were sufficient to give rise to significant amounts of newly generated neurons (Spalding et al., 2013). According to this study, 35% of hippocampal neurons are continuously turning over, forming a self-renewing population in the DG (Eriksson et al., 1998; Knoth et al., 2010; Spalding et al., 2013). Since the DG contains only slightly more than 35% of hippocampal neurons, nearly all its neurons must be subject to exchange in humans, with renewing rates of 1.75%/year (Spalding et al., 2013). These rates confirm that, in spite of possibly small populations of neuroblasts, the number of exchangeable neurons is high (700 new neurons/day) and allows an almost complete cell replacement in the granular layer within human lifespan (Spalding et al., 2013). In contrast, neurogenesis in rodents, although prominent among mammals (Amrein et al., 2011), displays only a 10% renewability rate of DG neurons during adulthood (Ninkovic et al., 2007; Imayoshi et al., 2008).

### Neurogenesis Throughout Age

These numbers demonstrate a remarkable extent of human neuron production, but turnover dynamics must be analyzed together with age-dependent changes for a complete understanding of hippocampal neurogenesis (Bergmann et al., 2015). In fact, in the early post-natal period, there is a general consensus that a dramatic and exponential decline in the population of cells with neuroblast markers occurs, comparatively to numbers seen during fetal development (Knoth et al., 2010; Spalding et al., 2013; Dennis et al., 2016; Sorrells et al., 2018). Afterwards, hippocampal dynamics are disputed.

As previously mentioned, some studies showed that DCX-positive neuroblasts may become undetectable after infancy, suggesting that neurogenesis ceases in the adult HF (Dennis et al., 2016; Sorrells et al., 2018).

Others found that neurogenesis occurs during adulthood and that the number of DCX-positive cells and the neuronal turnover rates (from the self-renewing fraction of HF neurons) present an insidious parallel decline along basal values (Knoth et al., 2010; Spalding et al., 2013), correlating with the neurogenic capacity of the DG (Spalding et al., 2013).

Finally, the most optimistic results were rather surprising, partially contradicting previous data from humans and consistent data from rodents and primates. Although a general decline in quiescent neural progenitors was observed in the DG, there was a stable number of double DCX-positive and PSA-NCAM-positive neuroblasts throughout aging (Boldrini et al., 2018). This suggests that a finite pool of early progenitors does not compromise the proliferative potential of this cell lineage as a whole, which may be supported in older age by late progenitors (Boldrini et al., 2018). Unfortunately, this study failed to analyze such cell markers in hippocampal samples from donors younger than 14 years of age, preventing a complete analysis for the total human age spectrum.

### Cell Count and HF Volume Dynamics

Neuroblast numbers and neuronal exchange rates could be associated with variations in the neuron count and HF volume, since the production of new cells could ultimately affect the number of neurons and the dimensions of this specific CNS region at each time point in human life. Indeed, there is evidence of a net loss of neurons in the whole HF with age, although stereological investigations have established that not all hippocampal subdivisions are equally affected (West, 1993; West et al., 1994; Simic et al., 1997). Neuroblasts occur in the DG and, interestingly, this region seems to be the least affected by the decrease in neuronal numbers (Bergmann et al., 2015). In fact, there might not be any actual change in the number of mature granule neurons in the DG (Boldrini et al., 2018), suggesting neurogenesis is an effective process in humans to oppose a basal rate of neuron depletion throughout aging. Curiously, there was also no age-related change in the volume of the DG in this study (Boldrini et al., 2018). However, others have shown that, in spite of its neurogenic potential, the human DG still presents a net loss of granule cells in adult life (Spalding et al., 2013) and that the total volume of the neuron-containing subdivisions of the HF, including the DG, decreases during adulthood (Simic et al., 1997; Daugherty et al., 2016), describing a scenario where neurogenesis is unable to prevent cell and volume depletion. The impact of adult neurogenesis on the total number of neurons in humans widely contrasts with the situation of rodents, where neurogenesis is additive and leads to an increase in granular layer volume and in the number of granule cells with age (Bayer, 1985). Nevertheless, the correlation between neurogenesis and hippocampal volume in humans is not yet entirely clarified.

Apart from these results, an increase is expected in the proportion of renewing neurons in the HF due to neurogenesis, since non-renewing cells die without being replaced (Spalding et al., 2013; Bergmann et al., 2015). The HF can be seen as a dynamic structure that permanently loses cells initially formed during development, with significant replacement in the DG with subgranular neuroblasts during adult life.

Further complexity in hippocampal cell dynamics is added by the fact that adult-generated cells are also lost during adulthood. In fact, within the renewing neuron population, younger cells were found to survive less than cells originated during development (Spalding et al., 2013). Unlike neurons from other hippocampal regions, which originated from development and are as old as the individual itself, the DG is composed of neurons generated at different time points throughout life, creating a complex mosaic where cell turnover is accompanied by a preferential loss of adult-generated neurons. This deepens the complexity of the HF neuron regulation and may possess relevance for the functional purposes of adult neurogenesis (Spalding et al., 2013).

## Regulatory Mechanisms for Adult Hippocampal Neurogenesis

Cell proliferation, neuronal differentiation and cell survival (Christie and Cameron, 2006) are responsible for the division of progenitor cells, selection of a neuronal fate rather than a glial one and incorporation and maintenance of new neurons into their final circuits, respectively. These three critical components of neurogenesis can be modulated, being subject to “control” and “regulation” by multiple factors (Kempermann, 2011; Aimone et al., 2014).

Both adult neurogenesis and developmental neurogenesis are tightly “controlled” by genetic and molecular programs, which grant a seemingly identical maturation process to adult-born neurons and neurons formed during embryogenesis. This explains the basis of the cytological organization seen in neuronal tissue (Kempermann, 2011; Aimone et al., 2014). On the other hand, there are “regulatory” intrinsic or extrinsic factors that could promote or suppress neurogenesis. These factors can depend on human behavior or the surrounding environment and, since they can actively up-regulate or down-regulate the formation of new neurons during adulthood, they are critical to understanding the relevance of adult neurogenesis on a functional basis (Kempermann, 2011), either behaviorally, cognitively or clinically.

Here, age, physical exercise and chronic stress will be addressed in more detail although several other factors should deserve similar attention, like diet (Cardoso et al., 2016) or sexual activity (Leuner et al., 2010).

### Age

As stated, there is a substantial decline in neuroblasts in the DG from infancy to adulthood (Knoth et al., 2010; Sanai et al., 2011), which contrasts with the uncompromised formation of new glial cells within this region (Capilla-Gonzalez et al., 2015). More studies are necessary to understand whether this decrease actually reaches zero (Dennis et al., 2016; Sorrells et al., 2018) or keeps basal values, with either low numbers of neuroblasts declining at prolonged rates (Kempermann, 2011) or considerable numbers of neuroblasts stable throughout the lifespan (Boldrini et al., 2018). It is essential to comprehend the mechanisms behind these unclear age-related changes, and understanding senescence at a neuroanatomical level could be necessary to distinguish physiological from pathological aging and the associated cognitive impairment (Capilla-Gonzalez et al., 2015).

For example, adding to the increased production of inflammatory factors, senescence of the cellular neurogenic niche may lead to a decrease in neurotrophic factors levels, turning the milieu less suitable for precursor cell activity (Lucin and Wyss-Coray, 2009; Aimone et al., 2014). Fibroblast growth factor-2 (FGF-2), insulin-like growth factor-1 (IGF-1) and vascular endothelial growth factor (VEGF) can induce proliferation of stem/progenitor cells in the DG in rodents and their levels decline considerably by middle age (Shetty et al., 2005), suggesting that age-related astrocytic changes could compromise neurogenesis (Bernal and Peterson, 2011). Restoration of FGF-2 and IGF-1 levels by intracerebroventricular infusion significantly promoted neurogenesis in adult rats (Lichtenwalner et al., 2001; Rai et al., 2007). Another regulator of neurogenesis brain-derived neurotrophic factor (BDNF), (Scharfman et al., 2005; Li et al., 2008; Schmidt and Duman, 2010), is highly associated to the induction of neuron survival and differentiation of hippocampal stem cells (Shetty and Turner, 1998) and presents decreased signaling in aging rodents accompanied by the decline in high-affinity tyrosine receptor kinase B (TrkB receptor) expression (Silhol et al., 2005). Although levels of hippocampal BDNF remain stable with age in rodents, serum BDNF is diminished in human adults and could mediate the age-related decline in hippocampal volume (Erickson et al., 2010).

### Physical Exercise

The neurogenic capacity of an adult brain can be enhanced, and the first evidence originated from a study where mice were housed in artificially enriched environments (Kempermann et al., 1997), granting sensory, social and motor stimulation (Aimone et al., 2014). By isolating the environmental variables, voluntary exercise was shown to be the primary condition behind the increased newborn neuron survival and upregulation in hippocampal neurogenesis (van Praag et al., 1999b; Ehninger and Kempermann, 2003).

It was shown that running increased cell division and enhanced survival and maturation of new cells on the HF (van Praag et al., 1999b; van Praag, 2008). Additionally, physical exercise was associated to DG plasticity, with a rise in long-term potentiation (LTP; van Praag et al., 1999a), and an increase of the complexity of the dendritic arborization and of the number of spines in granule cells (Eadie et al., 2005). Interestingly, LTP is more easily induced in younger neurons, due to different active and passive membrane properties (Schmidt-Hieber et al., 2004). Since exercise enhances LTP amplitude specifically in the DG (van Praag et al., 1999a), it is possible that newborn neurons could have an essential role in synaptic plasticity (Vivar et al., 2013; Duzel et al., 2016). These neuroplastic changes may present relevant implications, as exercise is known to facilitate learning and memory and to decrease depressive symptoms, which are linked to cognitive decline (Cotman et al., 2007; Hillman et al., 2008; Vivar et al., 2016).

Another remarkable alteration induced by aerobic training, inclusively seen in the adult human brain with magnetic resonance imaging (MRI), is the increase in hippocampal volume and the reversion of its 1%–2%/year decline (Erickson et al., 2011). A neuroprotective effect of physical exercise has been considered to fight brain atrophy and could protect against age- and disease-related mental decline (Cotman et al., 2007). This volume gain after physical exercise is apparently selective to the HF, which suggests that neurogenic cell proliferation could be at least partly responsible (Pajonk et al., 2010; Erickson et al., 2011; Biedermann et al., 2016). In mice, a strong correlation was found between the number of DCX-positive cells and the gray matter volume of the DG (Biedermann et al., 2016). Additionally, HF focal irradiation, which can nearly abolish neurogenesis, is sufficient to block the effects of running on hippocampal morphology (Fuss et al., 2014). Neurogenesis-related plasticity seems to play a role in exercise-induced volume changes, but other biological mechanisms could help explain such changes in the CNS (Ho et al., 2013), like dendritic arborization complexity (Eadie et al., 2005; Redila and Christie, 2006) or variations of the cerebral blood volume (Pereira et al., 2007).

**Figure 1:**
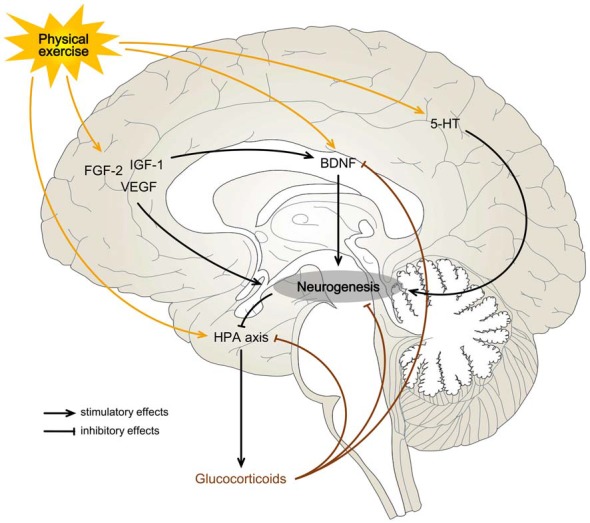
Possible mechanisms for exercise-induced effects on hippocampal neurogenesis. Physical exercise may be a rewarding or a stressful activity and can influence neurogenesis on the basis of a neuroendocrine balance. A predominant activation of the hypothalamic-pituitary-adrenocortical (HPA) axis, with a consequent increase in blood glucocorticoids (Droste et al., 2003), may overwhelm the stimulatory effects of exercise on neurogenesis by direct inhibition of the hippocampal progenitor cells or indirectly through brain-derived neurotrophic factor (BDNF) signaling blockage (Suri and Vaidya, 2013). When the hedonic component of a physical activity is more significant than the stress response, it may increase the levels of growth factors (BDNF, fibroblast growth factor-2 (FGF-2), insulin-like growth factor-1 (IGF-1) and vascular endothelial growth factor (VEGF)) and neurotransmitters (serotonin (5-HT)) in the hippocampal formation (HF) enough to promote neurogenesis (Gomez-Merino et al., 2001; Schoenfeld and Gould, 2012, 2013; Klempin et al., 2013).

Many cellular and molecular mechanisms could influence the positive effects of exercise in neurogenesis (Figure 1). Levels of BDNF, for instance, increase with exercise in humans (Ferris et al., 2007; Rasmussen et al., 2009) and gene and protein expression in the HF can be elevated after running in rodents (Neeper et al., 1995; Berchtold et al., 2001). Ablation of the gene encoding TrkB in precursor cells blocks the exercise-induced potentiation of neurogenesis (Li et al., 2008). Interestingly, mice lacking BDNF have impaired hippocampal LTP (Korte et al., 1995), which suggests this factor may be behind exercise-induced synaptic remodeling and plasticity (Park and Poo, 2013; Duzel et al., 2016). Like BDNF, neurotrophins FGF-2, IGF-1 and VEGF have been implicated in adult neurogenesis, and their levels are increased in blood after running, although recent studies have raised some doubts (Voss et al., 2013; Maass et al., 2016). Serum IGF-1 uptake by hippocampal neurons increases in response to exercise in rats, and administration of a blocking IGF-1 antiserum impaired exercise-induced stimulation of neurogenesis (Carro et al., 2000; Trejo et al., 2001). By blocking the IGF-1 receptor in the HF, the exercise-induced increase in BDNF was reversed, placing IGF-1 as a possible upstream molecule of the BDNF regulatory pathway (Ding et al., 2006). Consequently, peripheral IGF-1 may stimulate neurotrophic molecular cascades and influence synaptic plasticity (Vivar et al., 2013).

This regulatory influence of physical exercise could also be achieved through the blood. Exercise and the accompanying increase in cerebral blood flow and blood-brain barrier permeability may enhance the transportation of circulating factors into the brain (Sharma et al., 1991; Van der Borght et al., 2009; Aimone et al., 2014). Newborn hippocampal cells appear to be closely associated with blood vessels, forming angiogenic niches (Palmer et al., 2000). Interestingly, this microenvironment seems to be regulated by the circulating neurotrophic factors FGF-2 (Eppley et al., 1988), IGF-1 (Lopez-Lopez et al., 2004) and VEGF (Krum et al., 2002), and the two latter may play a central role in exercise (Lopez-Lopez et al., 2004; Bloor, 2005; Vivar et al., 2013). Like IGF-1, peripheral blockage of VEGF prevents the expected exercise-induced amplification of hippocampal neuroblasts in rodents (Fabel et al., 2003). VEGF may present a double action in this context, directly stimulating neural progenitor cells (Jin et al., 2002) and altering their vascular microenvironment (Fabel et al., 2003). Other evidence for this neurogenesis-angiogenesis correlation exists. Although there are contradicting results (Biedermann et al., 2016), studies have shown that, unlike in other hippocampal regions, the density of blood vessels in the DG rise in response to running in mice (Clark et al., 2009; Van der Borght et al., 2009). Physical activity selectively increases DG blood volume, both in mice and humans, and changes in this parameter correlated with post mortem evaluation of neurogenesis in rodents (Pereira et al., 2007). Indeed, the proliferation of neural progenitor cells may be dependent on the vasculature (Van der Borght et al., 2009).

Finally, other mechanisms, independent of the hippocampal vascular system, may additionally regulate the effects of exercise. For example, serotoninergic inputs may lead to an increased proliferation of granule cells and, during physical exercise, extracellular levels of this monoamine in the rat ventral HF are increased (Gomez-Merino et al., 2001). In mice lacking brain serotonin (5-hydroxytryptamine, 5-HT), activity-induced hippocampal proliferation was impaired (Klempin et al., 2013). The importance of 5-HT in adult neurogenesis goes far beyond physical activity since it might be fundamental to understand the pathophysiology of depression and its connection to environmental triggers and treatments (Mahar et al., 2014).

### Chronic Stress

Stress is one of the most studied modulators of neurogenesis in the mature brain. Like aging, stress leads to a general decrease in the neurogenic function of the HF (Schoenfeld and Gould, 2012) but, like physical exercise, it is a “regulatory” factor associated to behavioral activity and environmental stimuli (Kempermann, 2011). Expectedly, understanding mechanisms behind stress and its influence on the HF could be fundamental to study mental disorders.

The biological stress system is an adaptive mechanism that focuses the individual on detected environmental threats, either of psychological, physical or biological nature, and then attempts to regain homeostasis through neuroendocrine pathways (Lucassen et al., 2015). While acute stress is the consequence of a single stressful event, and can display physiological adaptive advantages, chronic stress is caused by multiple stressful events over a period of time (Aimone et al., 2014) and can be associated to the development of maladaptive responses (Aimone et al., 2014; Lucassen et al., 2015), like anxiety and depression (Mineur et al., 2006).

Uncontrollable and unpredictable chronic stress can lead to profound functional and morphological changes in the brain. In experimental animals subjected to stressful stimuli for extended periods, modification of presynaptic and postsynaptic structures occurs, with a decrease in hippocampal LTP (Pavlides et al., 2002) and dendritic arborization complexity (Magariños et al., 1996). These changes could affect the strength of excitatory synapses and information flow in the HF (Lucassen et al., 2014). Additionally, although conflicting results exist (Lucassen et al., 2014), evidence suggests that chronic stress could lead to a reduction in hippocampal volume in rats, although it might not be due to impaired neurogenesis (Schoenfeld et al., 2017). Finally, the decrease in the neurogenic capacity of the HF after chronic stressful experiences was demonstrated in tree shrews and rodents by several studies, using multiple stimuli like psychosocial subordination or electric shocks (Czeh et al., 2001; Dagyte et al., 2009). Methodological differences could justify the variability in results but, in overall, there is a compromise in cell proliferation, neuronal differentiation and survival (Schoenfeld and Gould, 2012).

Glucocorticoid hormones are traditionally viewed as the central mediators of these downregulatory effects (Schoenfeld and Gould, 2013). Stress involves both a rapid autonomic nervous system-mediated reaction and a delayed hypothalamic-pituitary-adrenocortical (HPA) axis-dependent response (Lucassen et al., 2015). The elevation of blood glucocorticoids allows a multi-systemic reaction that affects the brain and HF, where large amounts of glucocorticoid receptors (GR) exist (Wang Q. et al., 2013). In rats treated with corticosterone, there was a significant suppression of progenitor cell proliferation (Cameron and Gould, 1994; Brummelte and Galea, 2010), differentiation of newly-formed cells (Wong and Herbert, 2006) and post-mitotic survival (Wong and Herbert, 2004). In contrast, adrenalectomy stimulated hippocampal neurogenesis (Gould et al., 1992). Thus, stress-induced increases in glucocorticoids could be responsible for the stress-induced decrease in neurogenesis (Schoenfeld and Gould, 2012). Indeed, by blocking the HPA axis with mifepristone, a GR antagonist, the reduction in neurogenesis caused by chronic stressful stimuli was normalized (Oomen et al., 2007).

Despite the detrimental effect of stress and glucocorticoid hormones on neurogenesis, there are paradoxical situations (Egeland et al., 2015). Some behaviors and non-aversive activities increase circulating stress hormones but are instead associated with higher rates of adult neurogenesis (Schoenfeld and Gould, 2012). This is the case with voluntary physical exercise, where complex changes in the HPA axis increases glucocorticoid levels (Droste et al., 2003; Figure 1). Likewise, sexual experience elevates circulating stress hormones but seems associated with increases in cell proliferation in the rodent DG (Leuner et al., 2010). Therefore, the presence of elevated plasma levels of glucocorticoids may reflect the metabolic requirements of activated tissues and should not be used to distinguish the emotional component of a specific stimulus, since aversive psychosocial stressors and positive sexual experiences originate similar glucocorticoid responses (Buwalda et al., 2012). In this case, some mechanisms during exercise and sexual activity must protect the HF and allow neuronal growth despite HPA axis stimulation. Both exercise and sex have an intrinsic hedonic component and are generally rewarding experiences (Schoenfeld and Gould, 2012, 2013). Stimuli associated with a rewarding nature might unleash growth factors that counterbalance the effects of glucocorticoids, protecting the hippocampal neurogenic function (Schoenfeld and Gould, 2012, 2013). Some of these possible factors were previously mentioned, like BDNF, which is upregulated after running and sexual activity (Berchtold et al., 2001; Kim et al., 2013). This hypothesis could also help explain why extended voluntary running actually inhibits cell proliferation (Naylor et al., 2005). In fact, prolonged running, even voluntary, can develop into a stressor further activating the HPA axis, unbalancing the hormonal milieu and suppressing the positive effects of exercise on neurogenesis (Droste et al., 2003; Naylor et al., 2005; Lucassen et al., 2015). Additionally, part of the glucocorticoid-mediated decrease in neurogenesis and brain plasticity might also be caused by direct hormonal inhibition of the BDNF signaling pathway (Suri and Vaidya, 2013), which is known to decline in stressful experiences (Smith et al., 1995), thus establishing complex crosstalk between the two systems.

Other mechanisms may be responsible for stress-induced decreases in neurogenesis, like changes in neurotransmitter activity. Positive effects of 5-HT were shown when ablation of dorsal and medial raphe nuclei compromised serotoninergic input to the HF, resulting in a decrease of newly generated cells (Brezun and Daszuta, 2000a,b). The spontaneous firing activity of these 5-HT raphe neurons can be inhibited by chronic stressors in rats (Bambico et al., 2009), anticipating a possible neural pathway by which stress downregulates hippocampal neurogenesis. In fact, antidepressant treatment with a selective serotonin reuptake inhibitor (SSRI) induced increased levels of cell proliferation and reversed stress- and glucocorticoid-induced hippocampal changes (Malberg et al., 2000; Malberg and Duman, 2003; Qiu et al., 2007). These effects are representative of a more complex interconnection between monoaminergic transmission and stress-induced depressive disorders, placing neurogenesis in the center of a clinical paradigm.

## Functional and Clinical Correlates of Adult-Born Neurons in the HF

**Figure 2:**
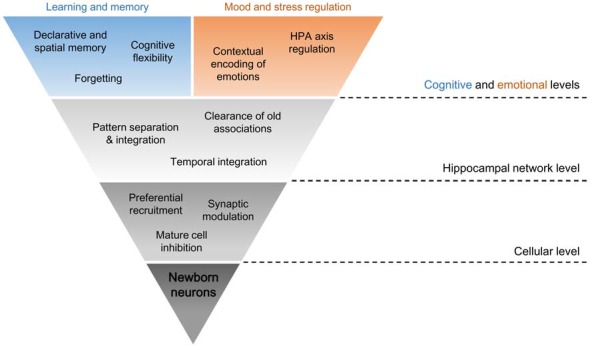
Dimensional levels of adult-born neuron functions in the HF. The distinct physiological properties of young adult-born hippocampal neurons sustain their specific interactions at a cellular level, like preferential recruitment of new inputs to these cells and changes to the remaining DG activity through mature cell inhibition and synaptic modulation. Such neurogenesis-induced intercellular connections inside the HF condition the neuronal circuitry as a whole, mainly in its role in context encoding of the surrounding environment and of new and old experiences. In other words, pattern separation, pattern integration, temporal integration and clearance of old associations in the network are modulated by neurogenesis. These hippocampal functions at the network level are necessary for some of the hippocampal functions at cognitive and emotional levels, which are fundamental for learning and memory and for mood and stress regulation. This might be a route by which ongoing neurogenesis may influence higher functions in humans.

The inherent methodological difficulty in directly evaluating hippocampal neurogenesis in human adults has led to the construction of hypotheses regarding the functional relevance of newborn neurons more based on theoretical considerations and less on direct evidence from studies in man. Therefore, questions remain whether neurogenesis is as paramount to humans as it apparently is for rodents. Although restrictive, this approach presents several correlations between human cognition or clinical conditions and neurogenesis-related behaviors or interventions that are worth exploring (Aimone et al., 2014; Mahar et al., 2014; Anacker and Hen, 2017). For organization purposes, functions attributable to new cells were divided into four groups (Figure 2).

### Functional Properties of Young Adult-Born Neurons

Newly-formed cells are not identical to mature granule cells. In their process of integration in the preexisting circuitry, many new neurons die within 4 weeks after birth (Kempermann et al., 2003), where others survive this critical period thanks to input-dependent and cell-specific neurotransmission (Tashiro et al., 2006). Initially, adult-born neurons display increased excitability, like reduced gamma-aminobutyric acid (GABA) inhibition, and enhanced plasticity, with a lower threshold for induction of LTP thanks to specific *N*-methyl-D-aspartate receptors (Wang et al., 2000; Ge et al., 2007). Despite the intrinsic excitability, immature cells also display reduced glutamatergic innervation and functional synaptic inhibition, possibly limiting spiking activity (Mongiat et al., 2009; Dieni et al., 2013). Conversely, mature granule cells present sparse activity due to hyperpolarized resting potentials and high tonic inhibition from local GABAergic interneurons (Jung and McNaughton, 1993; Houser, 2007). Surviving new cells will eventually present similar properties, inputs and firing behaviors as mature granule cells around 8 weeks after birth, but they still enter the hippocampal circuitry while transiently immature (Laplagne et al., 2006; Deng et al., 2010; Toni and Schinder, 2015). Since they also establish glutamatergic synapses with other hippocampal neurons (Toni et al., 2008), the introduction of their unique physiological cellular features in the neural network possibly contributes to learning and memory (Deng et al., 2010).

### The Cellular Level: Primary Interactions of New Neurons

Young adult-born neurons present primary interactions at a cellular level that could explain their remaining general functions in the HF, and that could sustain the basic principles of information processing in this region.

#### Preferential Recruitment

Taking advantage of the transitory hyperexcitability state (Anacker and Hen, 2017), it is hypothesized that new cells, when compared to mature granule cells, are preferentially recruited by new inputs arriving at the DG (Ramirez-Amaya et al., 2006; Kee et al., 2007; Marin-Burgin et al., 2012; Rangel et al., 2014). By studying immediate-early genes, whose expression is acutely increased by neuronal activity associated with learning and memory (Guzowski et al., 2005), it was found that young neurons were more active in processing new information (Kee et al., 2007). This differential selection is probably also explained by the inherent “silent activity” of mature granule cells (Jung and McNaughton, 1993; Aimone and Gage, 2011). Young neurons may bypass inhibitory control and have enough intrinsic excitability to compensate low excitatory inputs, outstanding as preferential for neuronal activity (Marin-Burgin et al., 2012). However, this “preferential recruitment” hypothesis has been contested by studies where granule cells, regardless of their developmental or adult origin, presented similar levels of activation (Jessberger and Kempermann, 2003; Stone et al., 2011; Dieni et al., 2013). Therefore, doubts remain whether excitatory inputs are high enough to trigger increased spiking activity in newborn neurons (Mongiat et al., 2009; Marin-Burgin et al., 2012; Dieni et al., 2013, 2016).

#### Synaptic Modulation

After genetically increasing the number of adult-born neurons in mice, a decrease in excitatory postsynaptic currents and spine density was seen in mature granule cells but without changing global measures of synaptic transmission (Adlaf et al., 2017). The proposed mechanism to explain these findings was “synaptic redistribution,” where synapses were not formed to accommodate new cells but instead were deviated from surrounding mature cells, possibly compensating initial weak excitatory inputs (Dieni et al., 2016; Adlaf et al., 2017). This strengthens reports suggesting that newborn neurons target afferent connections of mature neurons in a competitive process (Toni et al., 2007; Lacefield et al., 2012; McAvoy et al., 2016; Adlaf et al., 2017), eventually leading to a rewiring of the DG. Additionally, this could be a mechanism explaining the inverse relationship between the number of immature neurons and the excitability of the overall network (Ikrar et al., 2013), since competition from new cells might decrease input stimulation of mature cells and thus contribute to their sparse activity (Lacefield et al., 2012; Adlaf et al., 2017).

#### Mature Cell Inhibition

Another primary interaction new neurons developed in the DG is related to modulation of granule cell activity itself (Anacker and Hen, 2017). After optogenetic stimulation, it was found that new cells in the DG activate local GABAergic interneurons, which in turn inhibit mature granule cells (Drew et al., 2016). It also explains that reducing the number of new neurons allows an increase of overall excitability while enhancing hippocampal neurogenesis leads to a decrease in the strength of neuronal activation of the DG (Ikrar et al., 2013).

### The Hippocampal Network Level: Context Encoding

Considering the above-mentioned functions and interactions of immature adult-born neurons at a local cellular level, it is now necessary to understand their relevance as a distinct cellular population for the functioning of the DG.

#### Pattern Separation and Pattern Integration

Pattern separation is a network process responsible for the transformation of overlapping inputs into less similar outputs and has long been associated to the DG both in rodents (Gilbert et al., 2001) and humans (Bakker et al., 2008). In its role in forming distinct representations of different contexts, the HF uses pattern separation to minimize interference and to enhance discrimination when overlapping information is received from similar contexts, but not when they are substantially different (McHugh et al., 2007; Sahay et al., 2011b). Most inputs arrive from the entorhinal cortex and, in order to distinguish them, two non-mutually exclusive processes may occur: differential neuronal firing rates and recruitment of non-overlapping groups of neurons for each individual input (McAvoy et al., 2015). In concordance with the latter, since recipient granule cells outnumber afferent entorhinal cortex neurons, information is projected to a densely cellular “higher-dimension space” which facilitates discrimination (Deng et al., 2010). The DG tonic inhibition and low activity also contribute to the generation of sparse codes, where a small subset of active neurons is used for encoding (Wiskott et al., 2006). These are ideal to reduce overlap by facilitating recruitment of different granule cell populations, making each input represented in non-interfering small and distinct groups of cells (Jung and McNaughton, 1993; Leutgeb et al., 2007; Deng et al., 2010, 2013; McAvoy et al., 2015). Curiously, increasing the activity levels of the DG impairs formation of sparse patterns, disrupting context encoding (Kheirbek et al., 2013).

This highly tuned hippocampal mechanism of context encoding is also thought to depend on immature adult-born neurons. Mice subjected to hippocampal neurogenesis ablation had impaired spatial discrimination (Clelland et al., 2009) and disability in disambiguating two different contexts (Tronel et al., 2012). Conversely, inducible genetic expansion of the adult-born neuron population improved performance in differentiating overlapping contextual representations, making increased levels of neurogenesis sufficient for pattern separation improvement (Sahay et al., 2011a).

Several hypotheses have been proposed to explain these findings, and important correlations with functions at the cellular level exist. Young adult-born neurons may promote pattern separation by achieving a sparse activation of the DG (Sahay et al., 2011b; Aimone et al., 2014; McAvoy et al., 2015; Adlaf et al., 2017), considering synaptic redistribution and inhibitory feedback on mature cells. Ablating neurogenesis increases spontaneous network activity (Lacefield et al., 2012), making mature granule cells repeatedly available for responding to incoming inputs. This generates increasingly overlapping cellular representations for similar inputs, contrary to the functional properties the hippocampal network may need to separate and disambiguate patterns (Aimone et al., 2014).

**Figure 3:**
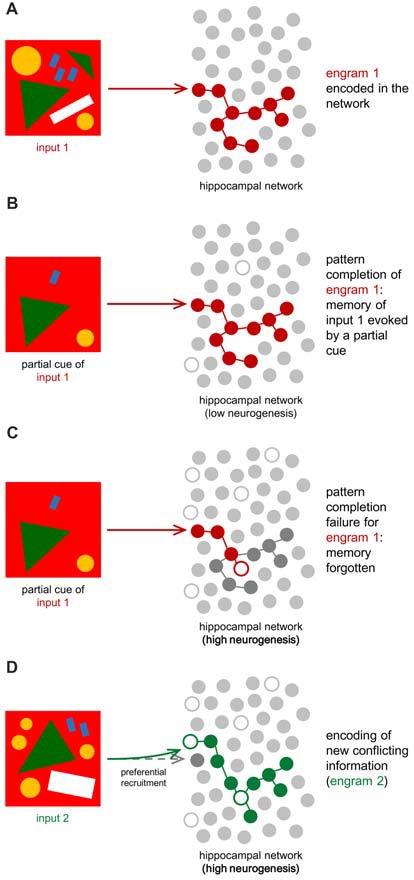
Hippocampal neuronal network for engram encoding. Oversimplification of the hippocampal network, defined by a layer of encoding units (gray circles). These do not illustrate neurons, but instead cellular representations for information encoding and engram storage. White circles stand for the added encoding units to the network (neurogenesis). **(A)** When initial information (input 1) arrives at the DG, it is represented in a specific pattern (engram 1, in red) now stored in the HF. **(B)** If neurogenesis is low, the hippocampal circuitry will be more stable and previously stored information can be recalled by partial cues evoking the correspondent engram, which was not affected by neurogenesis-dependent network remodeling (Frankland et al., 2013). **(C)** If neurogenesis is high, like during infancy, new neurons will alter the circuitry due to synaptic modulation and mature cell inhibition, changing the connections between encoding units (Frankland et al., 2013; Adlaf et al., 2017; Anacker and Hen, 2017). As a consequence, the same partial cue might not be enough to evoke the correspondent engram properly. If the engram was disrupted by neurogenesis, memory recall is compromised, and input 1 is forgotten. **(D)** When new information (input 2) is conflicting or similar to previous ones (example: a similar drawing but with a slightly different pattern), high neurogenesis may prevent the DG from creating identical engrams. In other words, two different yet similar inputs will be coded as different thanks to neurogenesis, which reinforces cellular representations available for pattern separation and facilitates recruitment of new cells. This leads to the formation of a second engram (engram 2, in green) different from the first one, in order to store input 2. According to the cognitive flexibility hypothesis, clearance of the old engram may also lead to the better encoding of the new one, reducing proactive interference (Epp et al., 2016; Anacker and Hen, 2017).

By adding encoding units, neurogenesis also increases the subset of different neurons available to create new codes in the DG. Their hyperexcitability additionally attracts new inputs to new cells, effectively separating two contexts in different hippocampal cellular representations (Aimone et al., 2014; Figure 3).

On the other hand, hyperexcitability is associated to a lower tuning specificity, making young neurons less suited for sparse encoding and more suited for responding broadly and indiscriminately at entorhinal inputs (Aimone et al., 2009; Danielson et al., 2016). Therefore, some hippocampal models place these immature cells at the frontline of “pattern integration,” encoding most features of the afferent impulse, identifying novel elements and/or associating similar events with each other (Aimone et al., 2009; Marin-Burgin et al., 2012; Kropff et al., 2015). Paradoxically, while responsive to a wide range of inputs, young neurons could benefit pattern separation in novel contexts, by supporting the association encoding of target stimuli with new features in the environment in a densely sampled representation, then highlighting details of the novel inputs to favor discrimination (Deng et al., 2010; Aimone et al., 2011; Kropff et al., 2015; Danielson et al., 2016).

Considering the hypothesis that new neurons might not present sufficient intrinsic excitability to compensate for the lack of glutamatergic inputs (Dieni et al., 2013), it is possible that they can actually benefit pattern separation through “low afferent sampling.” In this case, facing reduced synaptic connectivity, broad responsiveness is tampered, and young cells now sample a smaller fraction of entorhinal inputs than mature cells, encoding with high fidelity particular features of a given context with low overlap between representations of other neurons (Dieni et al., 2016).

#### Temporal Integration

Pattern separation and integration have been considered from a static perspective. However, neurogenesis is a dynamic process of continuous addition of new neurons to the DG, making time a critical variable. Considering their preferential recruitment, and since the immature granule cell population is in continuous change, new inputs are being received by continually different subsets of new neurons (Aimone et al., 2006). As a consequence, inputs separated by long timescales recruit different non-overlapping immature cells while closely-related inputs are encoded by the same newborn neuron population (Aimone et al., 2006; Rangel et al., 2014). In this way, neurogenesis could be sufficient for temporally proximal events to be coded as different thanks to pattern separation but with significant pattern overlap due to “temporal integration” associating them together (Aimone et al., 2006, 2014). Indeed, it was demonstrated in rats that colchicine-induced lesions to the DG, which also destroy immature cells, were capable of disrupting the formation of temporal associations for events occurring closer in time (Morris et al., 2013).

#### Clearance of Old Associations

If immature neurons present preferential recruitment, they should be expected to better encode novel information (Aimone et al., 2009), while mature neurons would remain responsible for older and familial information, possibly matching the environment at the time of their maturation (Aimone et al., 2009). With ongoing neurogenesis, integration of new neurons alters the circuitry through the development of multiple synaptic connections and through granule cell inhibition, as seen in the cellular level (Toni et al., 2007, 2008; Epp et al., 2011; Akers et al., 2014; Adlaf et al., 2017; Anacker and Hen, 2017). This, however, could potentially compromise recall of learned information, which depends on the same neuronal network that existed at the time of encoding (Frankland et al., 2013; Figure 3). The ability to recall patterns previously stored in the network by the detection of partial cues is called “pattern completion,” and it can be effectively impaired due to this network remodeling (Frankland et al., 2013; Akers et al., 2014). Computational models predict that new contexts are better-retained thanks to neurogenesis but that old patterns are affected by interference caused by the birth and integration of adult-born neurons (Weisz and Argibay, 2012). The proposed advantage of neurogenesis in this model could be easing the encoding of new information by weakening old conflicting information (Epp et al., 2016). “Proactive interference” is the name given when previously learned patterns preclude learning new ones with similar content. Neurogenesis can thus be regarded as a process that, when increased, leads to the better encoding of new information and disintegration of old associations, which reduces proactive interference but compromises pattern completion (Frankland et al., 2013). By decreasing integration of new adult-born neurons, pattern completion is preserved while proactive interference is increased (Frankland et al., 2013).

These proposed functions for neurogenesis at the hippocampal network level indeed contribute to context encoding of overlapping information. By enhancing neurogenesis, acquisition of conflicting information is facilitated by pattern separation (Sahay et al., 2011a) and temporal integration (Aimone et al., 2006), which ease distinction between spatially and temporally overlapping content, but also by destabilizing associations that are similar and already stored in the HF (Anacker and Hen, 2017). All this could lead to better adaptation to the new environment at both cognitive and emotional levels.

### The Cognitive Level: Learning and Memory

**Figure 4:**
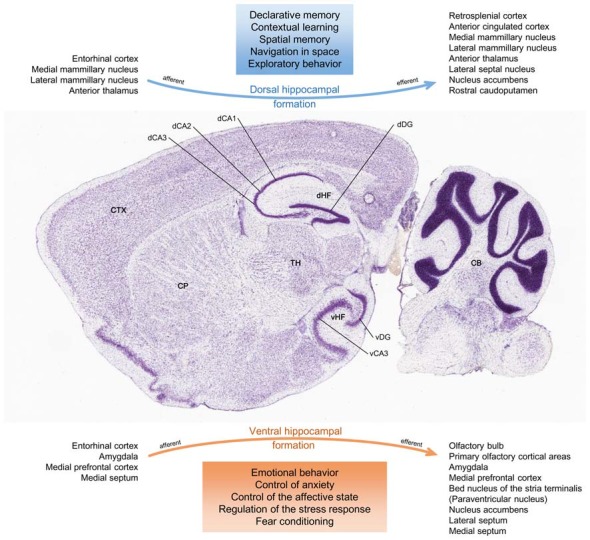
Functional divisions of the HF. Histological sagittal section of the adult mouse brain, intercepting the HF along its ventral-dorsal curvature. Accordingly to its anatomical configuration, the HF may be represented as a conjunction of two major networks with differential connections. The main afferent and efferent regions are seen in the figure. In this respect, there might be a functional segmentation where the dorsal HF mostly mediates cognitive processing of spatial information, memory and exploratory behavior, and where the ventral HF mainly coordinates responses to psychosocial stress and emotional experiences and regulates affective states (Fanselow and Dong, 2010; Tanti and Belzung, 2013; Anacker and Hen, 2017). CB, cerebellum; CP, caudoputamen; CTX, isocortex; dCA1, dorsal CA1 field; dCA2, dorsal CA2 field; dCA3, dorsal CA3 field; dDG, dorsal dentate gyrus granular layer; dHF, dorsal hippocampal formation; TH, thalamus; vCA3, ventral CA3 field; vDG, ventral dentate gyrus granular layer; vHF, ventral hippocampal formation. Image credit of the brain section: Allen Institute.

The HF presents a functional segmentation, where different gene expression and neural pathways separate a more anterior portion (ventral, in rodents) from a distinct posterior portion (dorsal, in rodents; Fanselow and Dong, 2010; Figure 4). Unlike its ventral counterpart, the dorsal HF primarily establishes cognitive functions and participates in declarative memory, spatial navigation and contextual learning (Fanselow and Dong, 2010; Kheirbek et al., 2013; Tanti and Belzung, 2013). Integrating young cells into these neuronal circuits is thought to influence hippocampal-dependent learning and memory (Deng et al., 2009; Wu and Hen, 2014).

#### Declarative and Spatial Memory

The HF contributes to integration and consolidation of declarative memory, a long-term process responsible for remembering events and facts. This role might be even more critical in the formation of the contextual component of these memories, through conjunctive encoding association of spatial and non-spatial information (Aimone et al., 2014; Kitamura and Inokuchi, 2014). Neurogenesis has been regarded as a process with a specific function here since it was shown that reducing the number of newly generated neurons compromised hippocampal-dependent forms of associative memory (Shors et al., 2001; Deng et al., 2010). Experimental observations have approached this hypothesis and the most common strategies focused on the effects of new neurons either before or after learning.

Neurogenesis is expected to present anterograde effects on memory, by which the addition of new neurons is significant before learning new contexts. For example, suppressing immature adult-born neurons in mice leads to a deficiency in forming long-lasting spatial memories and in adopting spatially precise learning strategies in mazes (Deng et al., 2009; Garthe et al., 2009). By depleting adult-born granule cells, overall results show impairments in cognitive performance, but results were inconsistent (Deng et al., 2010). Nevertheless, neurogenesis seems to be required for some hippocampal-dependent tasks, but not for hippocampal-independent ones (Deng et al., 2010). Pattern separation, for instance, not only is enhanced when neurogenesis is upregulated but also gives individuals greater capacity in distinguishing overlapping contextual representations (Sahay et al., 2011a). This benefits both episodic and spatial memory. Indeed, ablating neurogenesis in mice impairs association between multiple spatial cues in order to solve mazes, due to deficiencies in the pattern separation process and interference from similar spatial information (Dupret et al., 2008).

Conditioning neurogenesis after learning shows the retrograde effects on memory. New neurons are also fundamental for temporal integration in the HF and, by encoding temporally different contexts, these cells are well suited for separating contextual and episodic memories (Rangel et al., 2014). By preferentially ablating mature adult-born neurons after training specific tasks, memory expression was disrupted, and performance on those tasks was impaired, consistent with context and spatial memory deficits (Arruda-Carvalho et al., 2011). These results suggest that adult-born neurons become committed to memory traces and that neurogenesis is necessary to provide cellular representations called engrams for encoding novel information (Arruda-Carvalho et al., 2011; Anacker and Hen, 2017). This also points to the hypothesis that mature adult-generated granule cells respond preferentially to inputs they were exposed to during their immaturity and that, by specifically inhibiting these neurons, engram and memory reactivation is impaired (Tashiro et al., 2007; Anacker and Hen, 2017).

#### Forgetting

The hippocampal network clearance of old associations means that neurogenic stimuli change the neuronal circuitry in such a manner that previously effective cues to recall old memories can no longer trigger the corresponding stored pattern (Frankland et al., 2013), which was “forgotten” (Figure 3). However, since established memories may conflict with the integration of similar new ones, forgetting may be as crucial as memory and essential to react to the changing environment, allowing more efficient encoding of novel information (Frankland et al., 2013; Epp et al., 2016). Mice previously trained for spatial memory in a maze struggled to remember the solution of the same maze after exercise-induced neurogenesis but, remarkably, they were able to learn more readily the new solution when the maze was changed (Epp et al., 2016). This process seems to be specific for conflicting memories since neurogenesis did not facilitate encoding of associations not conflicting with the original learning (Epp et al., 2016).

#### Cognitive Flexibility

Cognitive flexibility is a process by which individuals use previous associations for learning when new challenges appear, allowing the selection of the most appropriate response (Kempermann, 2008; Burghardt et al., 2012). For instance, contextual memories are formed when mice learn the position where the shock zone on a rotating platform is located. Although ablating neurogenesis beforehand did not compromise learning this initial rule and finding the safety location, it impaired the ability to avoid a new shock zone when it was changed (Burghardt et al., 2012). Avoiding this new shock zone requires cognitive flexibility to suppress the initial response and to select a new behavior. If reversing a previously learned rule requires formation of adult-born neurons, then reduced levels of neurogenesis may be insufficient to prevent proactive interference between the old memory and the conflicting new information, in this case, the new location of the safety zone (Burghardt et al., 2012; Anacker and Hen, 2017). Pattern separation and clearance of old associations (by inhibiting established engrams) are responsible for reducing proactive interference and supporting cognitive flexibility during learning (Sahay et al., 2011a; Burghardt et al., 2012).

#### Integrating the Role of New Neurons on Learning and Memory

Neurogenesis can be regulated by learning itself (Gould et al., 1999; Kempermann, 2011). Although evidence has been contested, hippocampal-dependent learning about space and time seems to support integration and survival of immature granule cells during their critical period (Gould et al., 1999). This could be considered the starting point for the role adult neurogenesis plays in cognition. In other words, production of new neurons could be stimulated after learning, could promote forgetting of previous associations through competition with already incorporated neurons, and could facilitate new memory formation through coding of incoming inputs and subsequent consolidation of engrams, either in the HF or by transferring them to the cortex (Arruda-Carvalho et al., 2011; Burghardt et al., 2012; Josselyn and Frankland, 2012; Frankland et al., 2013; Kitamura and Inokuchi, 2014; Epp et al., 2016).

#### Cognitive Impairments With Age and Disease

Age itself has an exuberant influence on neurogenesis, making infancy and old age distinct periods of higher and lower rates of neuron formation, respectively (Knoth et al., 2010; Spalding et al., 2013). Curiously, humans and some rodents present “infantile amnesia,” a process that prevents the recall of declarative memories from early childhood. For example, human adults are unable to remember events from their first 2–3 years of life (Josselyn and Frankland, 2012). Neurogenesis has been proposed as a neurobiological substrate of infantile amnesia (Josselyn and Frankland, 2012), since the integration of elevated numbers of new neurons during the initial postnatal period may impede stability of the hippocampal memory storage (Frankland et al., 2013). As circumstantial evidence, the exponential decline of postdevelopmental neurogenesis occurs at the same time as the beginning of long-term memory retention (Josselyn and Frankland, 2012). Interestingly, using genetic and pharmacological approaches in infant mice, treatments that suppressed neurogenesis after memory formation reduced forgetting and, therefore, infantile amnesia (Akers et al., 2014). However, recent data suggest this may not be a process of memory erasure but rather of latent storage (Travaglia et al., 2016), leaving doubts on the role neurogenesis might play here.

The other extreme of the age spectrum also presents essential correlations with neurogenesis. Increasing age brings higher prevalence of cognitive decline and memory impairments, and these may parallel a progressive decline in neuroblast numbers (Bishop et al., 2010; Gil-Mohapel et al., 2013). Indeed, pattern separation is less efficient in older adults, leading to worse performances in recognition memory tasks (Toner et al., 2009). Using functional MRI scans, the same tasks revealed hippocampal hyperactivity in the elderly, which could be a sign of age-related modifications that impair the encoding mechanism of pattern separation (Yassa et al., 2011) and possibly related to changes in neurogenesis (Sahay et al., 2011b; Aimone et al., 2014). It is plausible that interventions that improve neurogenesis may be useful to treat hippocampal dysfunctions seen during normal aging (Sahay et al., 2011a). A behavioral intervention like physical exercise enhances neurogenesis and improves spatial memory and pattern separation in rodents (van Praag et al., 1999b; Creer et al., 2010). Running is also capable of reversing age-related memory impairments and neurogenesis decline (van Praag et al., 2005; Kronenberg et al., 2006; Cotman et al., 2007; Vivar et al., 2013; Duzel et al., 2016). A cause-effect relationship has not been established, but increasing neurogenesis may be behind some of the positive effects of exercise on learning, memory and general brain health, including in older individuals (van Praag, 2008; Sahay et al., 2011b; Duzel et al., 2016).

When aging becomes pathological and neurodegenerative diseases lead to progressive neuron depletion by the accumulation of misfolded proteins, cognitive decline and memory loss are substantially more severe (Kempermann, 2015). Alzheimer’s disease (AD) is a significant cause of dementia (Bishop et al., 2010) affecting the HF (Mu and Gage, 2011), and there might be a role for hippocampal neurogenesis in the pathophysiology of the disease. Indeed, transgenic animal models show dysfunctional adult-born neuron formation even before global neuronal loss (Winner et al., 2011) and proteins associated to the disease, like amyloid precursor protein and apolipoprotein E, were shown to regulate neurogenesis (Lazarov et al., 2010; Mu and Gage, 2011). Whether decreased neurogenesis is just a neuroanatomical manifestation of the disease or functionally contributes to memory impairment and cognitive decline is not known, and significant variability in experimental results prevents a clear correlation (Mu and Gage, 2011; Winner et al., 2011). Interestingly, in non-demented individuals with AD neuropathology, neural stem cells were preserved, when compared to symptomatic AD subjects, and correlated with cognitive capacity (Briley et al., 2016). Physical exercise can be proposed as a protective intervention for maintenance of neuroplasticity and cognition in AD, and neurogenesis can be a possible underlying mechanism (Mu and Gage, 2011; Duzel et al., 2016). Recent studies on neural stem cells also raise some hope for new effective treatments. In mouse models, intrahippocampal transplantation of human neural stem cells improved cognition by enhancing synaptogenesis (Ager et al., 2015), showing that the brain supports engraftment and differentiation of transplanted cells, even in aged subjects (Shetty and Hattiangady, 2016). These promising results highlight transplantation therapy as a possible intervention for AD (Apple et al., 2017b).

Overall, results on the interaction of adult-born neurons with neurodegenerative diseases are still controversial (Pino et al., 2017), but altered neurogenesis is a common finding (Winner and Winkler, 2015). Both Parkinson’s (PD) and Huntington’s disease (HD) frequently include cognitive impairment, and excessive α-synuclein, a hallmark protein in the pathophysiology of PD, leads to a decrease in hippocampal neurogenesis (Winner and Winkler, 2015), although it is likely that dopaminergic denervation also compromises precursor cell proliferation (Hoglinger et al., 2004). In HD, on the other hand, both hippocampal and striatal neurogenesis are reduced (Ernst et al., 2014; Winner and Winkler, 2015).

### The Emotional Level: Mood and Stress Regulation

The ventral HF plays significant roles in stress and modulation of emotional behavior, and it is thought to regulate the HPA axis (Fanselow and Dong, 2010; Kheirbek et al., 2013; Tanti and Belzung, 2013; Figure 4). Immature adult-born neurons also present this regional differentiation and, as a result, neurogenesis might actively participate in limbic functions and influence anxiety and depression by modulation of the stress response (Kheirbek et al., 2013; Wu and Hen, 2014).

#### Regulation of the HPA Axis

The ventral HF projects efferents to the bed nucleus of the stria terminalis, which in turn inhibits production of corticotropin-releasing hormone by the hypothalamic paraventricular nucleus. This hippocampal control of the HPA axis also presents feedback regulation, with glucocorticoids acting on the pituitary, hypothalamus, and HF (Anacker et al., 2011). Whether neurogenesis can be part of the hippocampal effects on the HPA axis is the missing link in this neuroendocrine chain. Indeed, by suppressing neurogenesis, the hormonal stress response is increased after exposure to a stressful stimulus and, afterwards, glucocorticoid levels present a slower recovery (Schloesser et al., 2009; Snyder et al., 2011). Neurogenesis might be necessary for the ventral HF to maintain inhibitory control over the hypothalamus and to regulate the usual endocrine response during stressful experiences, but not in basal conditions (Anacker and Hen, 2017). Predictably, when stress becomes pathological, hippocampal neurogenesis could be excessively inhibited, resulting in hyperactivity of the HPA axis with exaggerated responsiveness to future stress, propagating a negative cycle (Snyder et al., 2011).

#### Contextual Encoding of Emotions

Adult neurogenesis displays activity in pattern separation and clearance of old associations at the hippocampal network level, but their exact roles on the emotional regulation attributable to the HF are not fully understood. However, the HF is known to be involved in pattern separation of emotional information, and this neurogenic region uses emotional inputs to differentiate between similar experiences (Leal et al., 2014). Also, lesions in the basolateral amygdala suppress neurogenesis and prevent new neurons from responding to fear-conditioning tasks, suggesting a meaningful connection that provides emotional information to the HF (Kirby et al., 2012). An integrative view of cognition and emotion might then partly explain regulation of the stress response by adult-born granule cells in the HF. For example, when adult neurogenesis is down-regulated, pattern separation is impaired and old engrams are not cleared from the hippocampal network (Clelland et al., 2009; Frankland et al., 2013; Anacker and Hen, 2017). This leads to two significant consequences.

First, cognitive flexibility is unable to work correctly, and an increase in proactive interference with preservation of pattern completion is expected (Frankland et al., 2013). As a result, engrams of past stress-associated experiences are preserved in the hippocampal network. If that particular context no longer presents stressful stimuli, reduced numbers of newborn neurons may be insufficient to replace the previous stress-related association with current and conflicting information that the context is now safe (Anacker and Hen, 2017). Here, the perception of fear persists, and chronic stress may develop (Anacker and Hen, 2017). The immature granule cell population may thus be responsible for the extinction of fear memories seen in rodent experimental protocols (Deng et al., 2009).

The second consequence is that due to inefficient pattern separation, decreased neurogenesis leads to overgeneralization of external contexts, prompting the organism to respond in the same way to similar yet different stimuli (Sahay et al., 2011b). This lack of discriminatory capacity might be adaptive in stressful environments, where neurogenesis is downregulated, and generalization of all contexts as fearful leads to active avoidance of potential harms (Sahay et al., 2011b; Kheirbek et al., 2012). When maladaptive, like in chronic stress, this mechanism leads to overwhelmed stress responses facing innocuous new experiences, incorrectly perceived as aversive (Sahay et al., 2011b; Egeland et al., 2015). In this case, the stress response to the new stimulus is influenced by the negative emotional load of similar past experiences, giving new experiences a generalized stress-associated emotional context (Egeland et al., 2015).

#### Affective Regulation and Disease

Modulation of the stress response by adult-born neurons may be achieved by controlling the HPA axis and encoding emotional contexts and, when correctly regulated by adequate levels of neurogenesis, they may allow suitable affective states. If neurogenesis is suppressed, like during chronic stress, a vicious cycle may occur leading to continuous maladaptive stress responses and, possibly, reinforcing a disease state of chronic psychopathology like anxiety or depression (Gold, 2015; Anacker and Hen, 2017).

Anxiety-related behaviors may result from dysregulation of specific brain circuits. The ventral HF was shown to be responsible for some of these behaviors since DG granule cell activity suppresses innate anxiety (Kheirbek et al., 2013). Although ablation of new neurons does not affect basal levels of anxiety, animal models where neurogenesis was inhibited presented more often avoidance behaviors in the face of novel environments, denoting an increased negative impact of potentially threatening situations (Revest et al., 2009). These neurogenesis-related changes could reflect the impaired regulation of the HPA axis stress response (Snyder et al., 2011). They could also result from the cognitive impairment in the contextual encoding of emotions, with overgeneralization and incorrect assessment of risk-related information based on previously encountered aversive events, often associated to anxiety syndromes like post-traumatic stress disorder (PTSD; Revest et al., 2009; Lissek et al., 2010; Sumner et al., 2010; Sahay et al., 2011b; Kheirbek et al., 2012; Egeland et al., 2015).

Stress is also currently acknowledged as a causal factor in major depression, especially when psychological demands are higher than the capacity to cope with stressful stimuli (Mahar et al., 2014). Depression, as an heterogeneous disease, may have contributions to its etiology from multiple neural regions, and the HF has been one of the most extensively studied (Sahay and Hen, 2007). Macroscopically, MRI scans found decreased hippocampal volume in depressed subjects (Videbech and Ravnkilde, 2004), with the magnitude of this reduction correlating with illness duration in patients with multiple episodes (MacQueen et al., 2003). The possibility that hippocampal neurogenesis could help explain depression and its intrinsic changes to the CNS has prompted multiple studies and remains an open field of investigation.

The “neurogenic hypothesis” states that a reduced production of new DG cells is related to the pathophysiology of depression (Samuels and Hen, 2011) and several animal models of the disease have shown decreased neurogenesis (Sahay and Hen, 2007). In post mortem studies in humans, it was also found that untreated individuals with major depressive disorder presented smaller numbers of mature granule cells and reduced granular layer volume, specifically in the anterior HF (Boldrini et al., 2013). Additionally, younger age of disease onset was associated to even fewer granule neurons at observation (Boldrini et al., 2013). Since there was no apparent decrease in the number of neural progenitors in depressed patients (Boldrini et al., 2009), mood disorders could affect neurogenesis in the maturation and/or survival phases (Boldrini et al., 2013).

**Figure 5:**
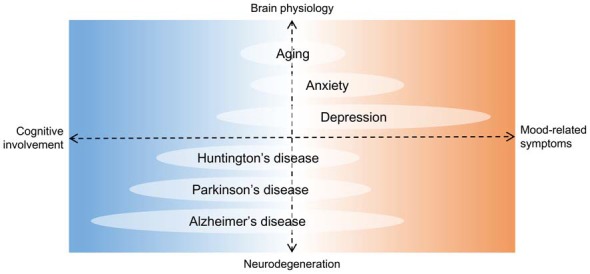
Hippocampal deficits in proposed neurogenesis-related conditions. Diagram showing aging and some diseases potentially associated with a chronic loss of adult newborn neurons in the HF. The horizontal axis is organized according to hippocampal deficits, with cognition and mood regulation at opposite ends. In the vertical axis, brain physiology and neurodegeneration are at opposite ends. Units are arbitrary. Although depression is the hallmark of affective disorders, it is intrinsically associated to cognitive decline. On the other hand, Alzheimer’s disease is a predominantly debilitating dementia but depressive or anxious symptoms are common. Therefore, regardless of the pathophysiology involved, no hippocampal condition can actually be separated in either of the deficit groups, and neurogenesis may be a mechanism underlying such clinical spectrum.

However, the central question here is whether decreased hippocampal neurogenesis is merely an epiphenomenon, a “neuroanatomical symptom” of depression, or is actually involved in hippocampal functional deficits seen in these patients, including not just mood dysregulation and inefficient responses to stress but also cognitive and memory impairments (Lee et al., 2013; Figure 5). On the one hand, although anhedonia-like behaviors have been provoked in neurogenesis-deficient mice (Snyder et al., 2011), ablating neurogenesis in healthy experimental animals mostly failed to induce depressive/anxious phenotypes, suggesting depletion of adult-born neurons in the HF is insufficient to trigger the complete pathophysiology of the disease (Petrik et al., 2012; Eliwa et al., 2017). On the other hand, experimental protocols studying chronic treatment with antidepressants in rodents showed an increase in hippocampal neurogenesis (Malberg et al., 2000), which might be necessary for their behavioral effects since abolishing neurogenesis blocks behavioral responses to medication in mice (Santarelli et al., 2003; Samuels and Hen, 2011). Pharmacologically-inducible neurogenesis may thus outweigh stress-induced hippocampal cell depletion and general atrophy (Malberg et al., 2000), possibly contributing to the positive effects of antidepressant therapy by reestablishing a functioning HF neuronal network at both emotional and cognitive levels. In spite of this, intact neurogenesis appears to be necessary for antidepressant efficacy only in some behavioral paradigms (David et al., 2009) and drug proliferative effects were dependent on the mice strain (Holick et al., 2008). This points to complementary neurogenesis-dependent and neurogenesis-independent actions and raises doubts on the need of an intact neurogenic HF for antidepressant treatment efficacy (Petrik et al., 2012).

This regulatory action of antidepressant drugs appears to occur during proliferation, maturation and survival of newborn neurons (Eliwa et al., 2017) and there is consistency between the latency of antidepressant effects and the duration of the maturation period for new cells (Sahay and Hen, 2007). Many drugs with antidepressant actions, or commonly used in depressed patients, have been studied from this neurogenic perspective.

Most antidepressants available today in clinical practice stimulate monoaminergic transmission in the CNS (Mahar et al., 2014; Eliwa et al., 2017). 5-HT, a neurotransmitter widely recognized as central in the pathophysiology of mood and anxiety disorders, is known to increase adult neurogenesis in mice and its blockage decreases proliferation of progenitor cells (Brezun and Daszuta, 1999; Schmitt et al., 2007; Apple et al., 2017a). Indeed, SSRI drugs selectively increase 5-HT levels, and chronic treatment with fluoxetine in rodents as showed reversion of hippocampal neurogenesis inhibition and reversion of behavioral changes associated to depressive states (David et al., 2009). In humans, SSRI-treated individuals with major depressive disorder presented greater amounts of amplifying neural progenitor cells than controls in the anterior HF (Boldrini et al., 2009) and, unlike untreated patients, had a granule cell count comparable to healthy subjects, with numbers that correlated well with DG volume in post mortem analysis (Boldrini et al., 2013). A possible reversion of hippocampal damage as a result of antidepressant therapy has also been seen in PTSD patients, where treatment with paroxetine lead to an increase in MRI-measured hippocampal volume and was effective in ameliorating symptoms and improving verbal declarative memory (Vermetten et al., 2003).

In an attempt to increase selectiveness of these serotoninergic drug effects, the 5-HT_1A_ receptor was studied and discovered to mediate fluoxetine’s effects in mice, both neurogenic and behavioral (Santarelli et al., 2003). The receptor is located postsynaptically in the HF and, remarkably, is associated to polymorphisms that may predispose to mental illnesses like anxiety and depression (Le François et al., 2008). Buspirone is commonly used for the treatment of generalized anxiety and in depressive states and, as a 5-HT_1A_ direct partial agonist, soon became a target of investigation in the field. Curiously, buspirone effectively enhances hippocampal neurogenesis in the mammalian brain (Grabiec et al., 2009). A drug with similar mechanism of action, tandospirone, also increases basal hippocampal neurogenesis dose-dependently (Mori et al., 2014) and prevents anxiety-related behavior provoked by psychosocial stress in rodents, while additionally protecting the DG from neurogenesis downregulation (Murata et al., 2015).

Other therapeutic options for depression include tricyclic drugs, which encompass multiple neurotransmitter modulating actions and, therefore, more side effects. Still, they remain in common clinical use. Imipramine, for example, was still effective in fluoxetine-resistant 5-HT_1A_ knock-out mice (Santarelli et al., 2003) and its influence on the noradrenergic system may be crucial, since noradrenalin also stimulates the early stages of neurogenesis (Eliwa et al., 2017). Treatment with imipramine can reverse neurogenic deficits in rat models of depression (Van Bokhoven et al., 2011).

If standard antidepressants fail to induce clinical remission, other therapeutic solutions must be proposed. This is the case with atypical antipsychotics, which can be considered in refractory major depressive disorder (Papakostas et al., 2007). Apart from being dopamine D_2_ receptor antagonists, they also convey modulating actions in serotoninergic and noradrenergic receptors. Possibly for this reason, clozapine promoted an increase in hippocampal neurogenesis and was effective in reverting stress-induced behavior in rodent models of depression, with additional positive results in behavioral flexibility tasks (Morais et al., 2017). Similarly, as an augmentation agent in fluoxetine-resistant rodents, quetiapine improved depressive behaviors while increasing the number of adult-born hippocampal neurons (Wang Y. et al., 2013). Clozapine and quetiapine are in great contrast to typical antipsychotics, like haloperidol, which is incapable of ameliorating depressive-like phenotypes in mice and, in fact, has negative effects on hippocampal neurogenesis (Morais et al., 2017).

By promoting a normal neurogenic function in the HF, drugs with antidepressant activity are seen as capable of reestablishing a more physiological neuronal network in depressed subjects. This may break the negative “vicious cycle,” with the HF regaining control over the stress response, and possibly the HPA axis. Chronic stress impairs the hippocampal-dependent inhibition of this endocrine pathway, generating hyperactivity alongside glucocorticoid hypersecretion, common in depressed patients (Nemeroff et al., 1984; Rubin et al., 1987; Surget et al., 2011; Zhu et al., 2014). These changes are relevant for disease progression since improved HPA axis regulation is associated with better outcomes in depression treatment and remission (Appelhof et al., 2006; Ising et al., 2007). At least part of the neurogenesis-dependent effects of antidepressant drugs might be achieved by recruiting new neurons to restore hippocampal-dependent inhibition of the HPA axis, thus enabling recovery (Surget et al., 2011; Eliwa et al., 2017).

Cognitive functions related to the hippocampal network might also play a role in affective regulation. Stress-related disorders and depressive-like traits are associated with a negative cognitive bias, where ambiguous situations are interpreted pessimistically (Enkel et al., 2010). Depression is also associated with a decrease in declarative memory and to worse performances on hippocampal-dependent tasks, including pattern separation (Dere et al., 2010; Shelton and Kirwan, 2013). Altogether, memory impairments and altered interpretation of ambiguous contexts may be related to a decrease in hippocampal neurogenesis (Gandy et al., 2017). Indeed, impaired cognitive flexibility and pattern separation inefficiency, which leads to overgeneralization (Sahay et al., 2011b), are commonly seen in depressed patients (Sumner et al., 2010; Belzung et al., 2015). The “neurogenic hypothesis” brings an enthusiastic but still remote possibility that modulating hippocampal neurogenesis could benefit cognitive impairments and aid recovery in depression (Sahay and Hen, 2007; Kheirbek et al., 2012; Eliwa et al., 2017). Chronic treatment with antidepressants might act in the full extent of the HF, not just in the “emotion-centered” anterior/ventral portion, possibly modulating the cognitive component of the stress response (Eliwa et al., 2017). However, central links are still missing here, specifically evidence on 5-HT and the reversal of decreased pattern separation with antidepressants in depressed subjects (Eliwa et al., 2017).

Just like mood disorders may compromise cognitive functions, neurodegenerative diseases are also prone to cause anxiety or depression-like symptoms (Winner and Winkler, 2015; Figure 5). This essential interplay between diseases of cognition and emotion may withstand as a “clinical argument” in favor of the integrative role for new neurons in the brain, as one of the mediators of hippocampal functional regulation of learning, memory, mood, and stress. Thus, conditions that affect the HF may be expected to perturb neurogenesis and possibly lead to both types of disability. Age itself is associated to cognitive and emotional dysregulation, and growing knowledge on the neurogenic effects of antidepressant drugs launched an important debate for future investigations in the field of healthy aging, among others (Boldrini et al., 2018).

## Conclusion

For the past half-century, adult neurogenesis has been extensively studied in an attempt to characterize the neurogenic areas of the mature brain and to determine its controlling and regulatory factors. Perhaps the most relevant question, that yet remains to be fully answered, is the one regarding the role new neurons play in the functional activity of the mature brain and whether these cells display any clinical relevance. There are still doubts on the extent of neuronal production in the human adult, and recent data (Dennis et al., 2016; Sorrells et al., 2018) has questioned the very existence of such a process. However, positive results identifying adult neurogenesis (Eriksson et al., 1998; Knoth et al., 2010; Spalding et al., 2013; Boldrini et al., 2018) cannot be entirely refuted and more optimistic studies, that enhance the role of adult-born neurons in numerous pathways (Aimone et al., 2014; Kempermann, 2015), contrast with the more skeptic view. So far, the greatest necessities in this field of science are more accurate approaches, cell markers and human imaging protocols that can efficiently study neurogenesis and reconcile discrepant results. Although contradictory evidence exist, the proposed hypothesis and subsequent clinical correlates represent the ongoing research and are essential to understanding one of the possible neuroplastic events that ensures the continuous modification of the CNS during adulthood.

## Author Contributions

PB made the initial search of articles and the first draft of the manuscript. JA was the mentor and corrected the several versions of the manuscript.

## Conflict of Interest Statement

The authors declare that the research was conducted in the absence of any commercial or financial relationships that could be construed as a potential conflict of interest.

## Abbreviations

5-HT: 5-hydroxytryptamine, serotonin
AD: Alzheimer’s disease
BDNF: brain-derived neurotrophic factor
BrdU: bromodeoxyuridine (thymidine analog)
CNS: central nervous system
DCX: doublecortin (neuroblast marker)
DG: dentate gyrus
FGF-2: fibroblast growth factor-2
GABA: gamma-aminobutyric acid
GR: glucocorticoid receptors
HD: Huntington’s disease
HF: hippocampal formation
HPA: hypothalamic-pituitary-adrenocortical axis
IGF-1: insulin-like growth factor-1
LTP: long-term potentiation
MRI: magnetic resonance imaging
PD: Parkinson’s disease
PSA-NCAM: polysialylated neural cell adhesion molecule (migratory cell marker)
PTSD: post-traumatic stress disorder
RMS: rostral migratory stream
SGZ: subgranular zone, dentate gyrus, hippocampal formation
SSRI: selective serotonin reuptake inhibitor (antidepressant group)
SVZ: subventricular zone
TrkB: tyrosine receptor kinase B (high-affinity receptor for BDNF)
VEGF: vascular endothelial growth factor.
